# Preexisting morbidity profile of women newly diagnosed with breast cancer in sub‐Saharan Africa: African Breast Cancer—Disparities in Outcomes study

**DOI:** 10.1002/ijc.33387

**Published:** 2020-11-25

**Authors:** Oluwatosin A. Ayeni, Shane A. Norris, Maureen Joffe, Herbert Cubasch, Moses Galukande, Annelle Zietsman, Groesbeck Parham, Charles Adisa, Angelica Anele, Joachim Schüz, Benjamin O. Anderson, Milena Foerster, Isabel dos Santos Silva, Valerie A. McCormack

**Affiliations:** ^1^ SAMRC/Wits Developmental Pathways for Health Research Unit, Department of Paediatrics, Faculty of Health Sciences University of the Witwatersrand Johannesburg Gauteng South Africa; ^2^ Noncommunicable Diseases Research Division Wits Health Consortium (PTY) Ltd Johannesburg Gauteng South Africa; ^3^ Department of Surgery, Chris Hani Baragwanath Academic Hospital and Faculty of Health Sciences University of Witwatersrand Johannesburg Gauteng South Africa; ^4^ Department of Surgery Makerere University Kampala Uganda; ^5^ Department of Oncology Windhoek Central Hospital Windhoek Namibia; ^6^ Department of Obstetrics and Gynecology University of North Carolina Chapel Hill North Carolina USA; ^7^ Department of Surgery Abia State University Teaching Hospital Aba Nigeria; ^8^ Department of Surgery Federal Medical Centre Owerri Nigeria; ^9^ Section of Environment and Radiation International Agency for Research on Cancer, (IARC/WHO) Lyon France; ^10^ Fred Hutchinson Cancer Research Center Seattle Washington USA; ^11^ Department of Non‐communicable Disease Epidemiology London School of Hygiene & Tropical Medicine London UK

**Keywords:** breast cancer, chronic condition, multimorbidity, stage at diagnosis and sub‐Saharan Africa

## Abstract

The presence of preexisting morbidities poses a challenge to cancer patient care. There is little information on the profile and prevalence of multi‐morbidities in breast cancer patients across middle income countries (MIC) to lower income countries (LIC) in sub‐Saharan Africa (SSA). The African Breast Cancer–Disparities in Outcomes (ABC‐DO) breast cancer cohort spans upper MICs South Africa and Namibia, lower MICs Zambia and Nigeria and LIC Uganda. At cancer diagnosis, seven morbidities were assessed: obesity, hypertension, diabetes, asthma/chronic obstructive pulmonary disease, heart disease, tuberculosis and HIV. Logistic regression models were used to assess determinants of morbidities and the influence of morbidities on advanced stage (stage III/IV) breast cancer diagnosis. Among 2189 women, morbidity prevalence was the highest for obesity (35%, country‐specific range 15‐57%), hypertension (32%, 15‐51%) and HIV (16%, 2‐26%) then for diabetes (7%, 4%‐10%), asthma (4%, 2%‐10%), tuberculosis (4%, 0%‐8%) and heart disease (3%, 1%‐7%). Obesity and hypertension were more common in upper MICs and in higher socioeconomic groups. Overall, 27% of women had at least two preexisting morbidities. Older women were more likely to have obesity (odds ratio: 1.09 per 10 years, 95% CI 1.01‐1.18), hypertension (1.98, 1.81‐2.17), diabetes (1.51, 1.32‐1.74) and heart disease (1.69, 1.37‐2.09) and were less likely to be HIV positive (0.64, 0.58‐0.71). Multi‐morbidity was not associated with stage at diagnosis, with the exception of earlier stage in obese and hypertensive women. Breast cancer patients in higher income countries and higher social groups in SSA face the additional burden of preexisting non‐communicable diseases, particularly obesity and hypertension, exacerbated by HIV in Southern/Eastern Africa.

## Abstract 2

What's new?

Patients who get cancer on top of other health conditions can be challenging to treat. Here, the authors analyze the prevalence of chronic conditions in women newly diagnosed with breast cancer in sub‐Saharan Africa. They looked at obesity, hypertension, diabetes, asthma/COPD, heart disease, tuberculosis, and HIV. Overall, 27% of the women had 2 pre‐existing conditions. Obesity boosts breast cancer risk, and obesity rates are rising among higher income groups. HIV is a problem in the southern African populations, and while HIV is not thought to contribute to breast cancer, reports suggest that HIV‐positive patients have poorer prognosis.

**Abbreviations**
ABC‐DO: Africa Breast Cancer—Disparities in Outcomes
BMI: body mass index
CD: communicable diseases
CI: confidence interval
COPD: chronic obstructive pulmonary disease
ELISA: enzyme‐linked immunosorbent assay
HICs: high‐income countries
HIV: human immunodeficiency virus
LMICs: low and middle income countries
NCDs: non‐communicable diseases
OR: odds ratio
SEP: socioeconomic position
SSA: Sub‐Saharan Africa
TNM: tumour, node, metastasis classification

## 1 INTRODUCTION

Breast cancer is the most common malignancy and the leading cause of cancer mortality in women worldwide. It is also the most common cancer among women in sub‐Saharan Africa (SSA),^1^ where the incidence of this cancer is on the rise. These temporal changes form part of an epidemiological transition occurring in SSA, characterised by improved control of infectious diseases, ageing populations and increasing prevalence of risk factors for non‐communicable diseases (NCD) due to economic advancement, urbanisation and lifestyle modifications.^2^ These syndemics also result in multimorbidity (the co‐occurrence of two or more chronic comorbidities in one person)^3^ at the individual level, including in breast cancer patients.

Survival after breast cancer diagnosis is determined by patient characteristics and disease‐related factors such as stage at diagnosis and breast cancer subtypes.^4^ In addition to these factors, studies mostly originating from high‐income countries (HICs) have shown that several preexisting morbidities are associated with lower breast cancer survival, poorer overall outcomes and they also impact on treatment recommendations and tolerance.^5^, ^6^ These morbidities include obesity, diabetes and insulin resistance, stroke, body composition and mental health illnesses, that is, conditions that tend to be more common in older breast cancer patients.^7^

Preexisting chronic conditions have also been linked to stage at cancer diagnosis, but the direction, degree and mechanisms of associations have not been consistent.^8^, ^9^, ^10^ The presence of preexisting morbidities have been linked to increased risk of metastatic disease at diagnosis,^8^ whereas links to earlier stage at diagnosis have been reported in settings served by population‐based breast cancer screening.^11^ Some studies also noted earlier stage at diagnosis in women with hypertension,^9^, ^12^ possibly arising from a “surveillance effect” (ie, greater clinical scrutiny upon follow‐up for hypertension/greater opportunity to seek help during health system contacts for other illnesses).

In the SSA setting, there is a paucity of data on the profile of preexisting morbidity profiles among breast cancer patients. Such profiles are likely to differ across SSA countries at differing stages of economic, developmental and lifestyle transitions, that is, different in SSA middle income countries (MICs) from low income countries (LICs).^13^, ^14^ Furthermore, in addition to the aforementioned conditions, the setting‐pertinent infectious disease of HIV needs to be taken into account due to the ageing HIV‐positive population successfully treated with antiretroviral drugs.^15^ Establishing these SSA‐specific morbidity profiles of breast cancer patients will form a first step to inform priorities for feasible management strategies in constrained public health systems.

To date, we have investigated this morbidity profile among breast cancer patients diagnosed at five hospitals in South Africa and found that 44% of women had preexisting morbidities at diagnosis: 53% were obese, 41% hypertensive, 22% HIV positive and 14% were diabetic. Multimorbidities were linked to older age and higher socioeconomic status.^12^ In the present study, we broadened our perspective to five SSA countries, including the upper MICs of South Africa and Namibia, lower MICs of Zambia and Nigeria and LIC of Uganda. Our aims were 3‐fold: (a) to describe the prevalence and profile of preexisting chronic conditions and multimorbidity (at least two chronic conditions in addition to breast cancer) in women newly diagnosed with breast cancer in five countries in SSA; (b) to determine the sociodemographic factors associated with individual chronic conditions and multimorbidity and (c) to assess whether any of the individual chronic conditions or multimorbidity was associated with breast cancer stage at diagnosis.

## 2 METHODS

### 2.1 Study design and study setting

Presence of preexisting chronic conditions and multimorbidity in women newly diagnosed with breast cancer was examined within the African Breast Cancer—Disparities in Outcomes (ABC‐DO) study cohort, a prospective multi‐country hospital‐based breast cancer cohort in Southern (South Africa, Namibia and Zambia), Western (Nigeria) and Eastern (Uganda) SSA countries.^16^ Incident breast cancer patients were recruited at Windhoek Central Hospital, Namibia; Abia State University Teaching Hospital and the Maranatha Private Clinic, Aba and the Federal Medical Centre, Owerri, Nigeria; Chris Hani Baragwanath Academic Hospital, Soweto, South Africa; Mulago Hospital and the Uganda Cancer Institute, Kampala, Uganda; and the Cancer Diseases Hospital and University Teaching Hospital, Lusaka, Zambia. The ABC‐DO protocol was previously published.^16^

### 2.2 Participants

Recruitment commenced from September through December 2014 in most centres and was completed by April 2017 except in Zambia (May 2016 through September 2017). Women ≥18 years of age with a clinically/histologically confirmed newly diagnosed breast cancer who provided written consent to participate in the study and provided access to their medical records and tumour tissue were enrolled. ABC‐DO was approved by ethics committees at each recruitment institution: IARC (IEC 13‐19, IEC15‐18), the London School of Hygiene and Tropical Medicine (6459), University of Witwatersrand, South Africa (M150345), Council for Science and Technology (HS 1588) and the Ministry of Health and Social Services of Namibia (17/3/3), University of Zambia Biomedical Research Ethics Committee (004‐08‐15), Federal Medical Centre Owerri, Abia State University Teaching Hospital and Uganda National Council for Science and Technology (HS 1588). Our study was performed in accordance with the Declaration of Helsinki.

The present analyses of preexisting morbidities were conducted overall and by site‐race group, with Namibian women separated into black and non‐black ethnic groups as these groups were different in socioeconomic profile and stage distribution.^17^ Participants from other countries were almost exclusively black women, 42 non‐black South Africans were excluded from the analysis as this group was too small to analyse separately.

### 2.3 Exposure and outcome data

The same face‐to‐face baseline questionnaire was utilised in all hospitals except in South Africa where a preexisting survey instrument was administered and harmonised to ABC‐DO specifications. For this analysis, we included questions on age, self‐identified ethnic group and marital status. Educational level was categorised as primary education or none and secondary education or higher. We grouped employment into two categories, employed (highly skilled/skilled, employed in South Africa) vs unemployed (unskilled, unemployed and retired in South Africa). A score for socioeconomic position (SEP) was generated from self‐reported household facilities and possessions: home ownership, flush toilet, indoor running water, vehicle, electricity, gas or electric stove, refrigerator, landline phone and a bed. A score of +1 was allocated to each possession ranging from 0 (low) to 9 (high).

We examined the presence of seven chronic conditions: obesity, hypertension, diabetes, heart disease, asthma/chronic obstructive pulmonary disease (COPD), HIV infection and tuberculosis. The seven conditions were included because of their known association with breast cancer prognosis (obesity,^18^ diabetes,^19^ HIV^4^), their known impact on treatment (eg, cardiotoxicity and worsening of hypertension^20^) and/or their known high prevalence in the least parts of SSA where regular treatment may provide an avenue for early cancer detection (eg, HIV and tuberculosis,^21^ COPD,^22^ hypertension and heart disease^23^). Body weight and height were measured at enrolment, and obesity was defined as a body mass index (BMI) ≥30.0 kg/m^2^. Patients were asked if they had ever been treated for tuberculosis and whether they had ever been diagnosed and treated with hypertension, diabetes, heart disease, asthma/COPD (questionnaire on chronic conditions collected provided as Supplementary material). HIV infection (yes vs no/not known) was based on self‐reports, with 90% of the cohort (apart from South Africa) reporting the later; however, there was a 97% agreement with clinical records among those reported negative. In South Africa, HIV status was tested as part of the diagnostic workup, using the enzyme‐linked immunosorbent assay by the National Health Laboratory Services. We defined multimorbidity as having ≥2 of these seven chronic conditions, in addition to breast cancer.

Breast cancer stage at diagnosis was determined using the American Joint Committee on Cancer (AJCC) TNM staging system^24^ and categorised for analyses as early (Stages I and II) and advanced (Stages III and IV).

### 2.4 Statistical methods

We described the pattern and prevalence of each chronic condition by site‐race group using Pearson's chi‐squared and Fisher's exact tests. We examined associations of sociodemographic factors with each chronic condition and with multimorbidity (≥2 of these chronic conditions in addition to the breast cancer) using logistic regression analysis. Logistic regression was also used to examine whether each chronic condition and multimorbidity were associated with advanced stage breast cancer (Stages III and IV), as compared to early‐stage breast cancer (Stages I and II). Variables for which *P* values were <0.1 in bivariate analysis with advanced stage breast cancer were evaluated and ORs were assessed adjusting for each covariate, HIV status, age and country (as a proxy for level of health care). Analysis was performed using Stata version 15 (StataCorp Ltd, TX).

## 3 RESULTS

A total of 2189 women were included as follows: 720 (33%) from South Africa, 478 (22%) (99 non‐black and 379 black) from Namibia, 187 (9%) from Zambia, 382 (17%) from Nigeria and 422 (19%) from Uganda (Table 1). The mean age at diagnosis was 51.7 ± 13.9, with women from Zambia, Nigeria and Uganda (range of mean age 48.4‐49.4 years) significantly younger than women from Namibia and South Africa (range of mean age 52.5‐57.1 years). Overall 62% of the women had at least secondary school education with the non‐black Namibian (87%), South African (77%) and Nigerian (72%) women more likely to have had at least secondary education compared to black Namibian (49%), Zambian (47%) and Ugandan (42%) women. In keeping with these low and middle income (LMIC) settings, most women had low to medium SEP with higher SEP in non‐black Namibia women. Smoking prevalence was low, ranging from <1% in Nigeria to 43% among the non‐black Namibian women. Approximately 59% of the women presented with advanced stage breast cancer overall (50% in South Africa to 75% in Nigeria), with the exception of non‐black Namibian women where the majority presented with early‐stage disease (78%) (Table 1).

**TABLE 1:** Characteristics of women newly diagnosed with breast cancer in sub‐Saharan Africa, by country and race; African Breast Cancer—Disparities in Outcomes study

	Southern Africa (SA)				West Africa	East Africa	
	South Africa	Namibia non‐Black	Namibia Black	Zambia	Nigeria	Uganda	Total
	N = 720 (%)	N = 99 (%)	N = 379 (%)	N = 187 (%)	N = 382 (%)	N = 422 (%)	N = 2189 (%)
Age in years, mean ± SD	54.5 ± 14.3	57.1 ± 12.4	52.5 ± 14.6	49.4 ± 14.2	48.8 ± 12.2	48.4 ± 12.6	51.7 ± 13.9
BMI (kg/m^2^)	31.7 ± 7.7	29.1 ± 6.8	26.4 ± 6.7	26.5 ± 6.3	26.4 ± 5.7	25.6 ± 4.6	28.1 ± 7.0
Marital status							
Married	233 (32.4)	58 (58.6)	122 (32.2)	110 (58.8)	250 (65.4)	199 (47.2)	972 (44.4)
Not married	487 (67.6)	41 (41.4)	257 (67.8)	77 (41.2)	132 (34.6)	223 (52.8)	1217 (55.6)
Received secondary education							
Yes	551 (76.5)	86 (86.9)	184 (48.5)	87 (46.5)	276 (72.3)	178 (42.2)	1362 (62.2)
No	169 (23.5)	13 (13.1)	195 (51.5)	100 (53.5)	106 (27.7)	244 (57.8)	827 (37.8)
Employment status							
Employed	202 (28.1)	98 (99.0)	346 (91.3)	73 (39.0)	357 (93.5)	331 (78.4)	1407 (64.3)
Unemployed	518 (71.9)	1 (1.0)	33 (8.7)	114 (61.0)	25 (6.5)	91 (21.6)	782 (35.7)
SEP							
Low (0–3)	273 (37.9)	0 (0.0)	166 (43.8)	67 (35.8)	161 (42.1)	249 (59.0)	916 (41.8)
Medium (4–6)	433 (60.1)	39 (39.4)	130 (34.3)	69 (36.9)	167 (43.7)	90 (21.3)	928 (42.4)
High (7–9)	14 (1.9)	60 (60.6)	83 (21.9)	51 (27.3)	54 (14.1)	83 (19.7)	345 (15.8)
Ever consumed alcohol?							
No	565 (78.5)	37 (37.4)	185 (48.8)	123 (65.8)	178 (46.6)	225 (53.3)	1313 (60)
Yes	155 (21.5)	62 (62.6)	194 (51.2)	64 (34.2)	204 (53.4)	197 (46.7)	876 (40)
Ever smoked?							
No	677 (94.0)	56 (56.6)	324 (85.5)	185 (98.9)	381 (99.7)	408 (96.7)	2031 (92.8)
Yes	43 (6.0)	43 (43.4)	55 (14.5)	2 (1.1)	1 (0.3)	14 (3.3)	158 (7.2)
Stage at diagnosis							
Stages I and II	347 (49.6)	77 (77.8)	139 (36.7)	60 (40.3)	88 (25.1)	142 (36.5)	853 (41.3)
Stages III and IV	353 (50.4)	22 (22.2)	240 (63.3)	89 (59.7)	262 (74.9)	247 (63.5)	1213 (58.7)

*Note*: Missing values for covariates were as follows: stage at diagnosis (n = 132).

Abbreviations: BMI, body mass index; SEP, socioeconomic position.

### 3.1 Prevalence of chronic conditions and multimorbidity

**FIGURE 1:**
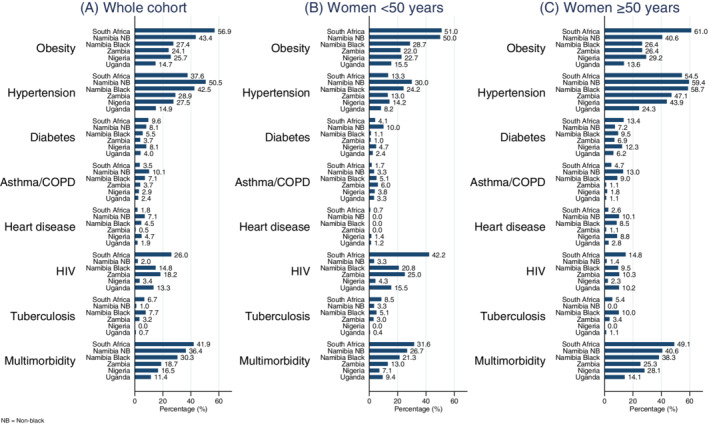
The prevalence of preexisting chronic conditions at breast cancer diagnosis in the African Breast Cancer—Disparities in Outcomes study, overall and by age at cancer diagnosis. Multimorbidity prevalence indicates the presence of two or more of the seven preexisting chronic condition at the time of breast cancer diagnosis [Color figure can be viewed at wileyonlinelibrary.com]

Hypertension and obesity dominated as the most common chronic conditions in breast cancer patients in each country, with hypertension ranking first in all countries except in South Africa where obesity ranked as the most common preexisting chronic condition. HIV ranked third prevalent in all countries except in non‐black Namibians (where asthma/COPD was in third rank) and in Nigeria (diabetes ranked third). Diabetes was the fourth most prevalent chronic condition in most countries except among the black Namibian women (tuberculosis was fourth) and in Nigeria (heart disease was fourth) (Table 2). Overall South Africa had the highest prevalence of HIV in the whole cohort (26%) (Table 2) with 42% of the women <50 years of age infected with HIV (Figure 1).

**TABLE 2:** Prevalence of preexisting chronic conditions in women newly diagnosed with breast cancer in sub‐Saharan Africa, by country site; African Breast Cancer—Disparities in Outcomes study

	Southern Africa (S.A.) Upper MICs			S.A. Lower MIC	West Africa Lower MIC	East Africa LIC	
	South Africa	Namibia Non‐Black	Namibia Black	Zambia	Nigeria	Uganda	Total
Ranked chronic conditions	N = 720 (%)	N = 99 (%)	N = 379 (%)	N = 187 (%)	N = 382 (%)	N = 422 (%)	N = 2189 (%)
Most common	Obesity (57)	Hypertension (51)	Hypertension (43)	Hypertension (29)	Hypertension (28)	Hypertension (15)	Obesity (35)
Second most common	Hypertension (38)	Obesity (43)	Obesity (27)	Obesity (24)	Obesity (26)	Obesity (15)	Hypertension (32)
Third most common	HIV (26)	Asthma/COPD (10)	HIV (15)	HIV (18)	Diabetes (8)	HIV (13)	HIV (16)
Fourth most common	Diabetes (10)	Diabetes (8)	Tuberculosis (8)	Diabetes (4)	Heart disease (5)	Diabetes (4)	Diabetes (7)
Fifth most common	Tuberculosis (7)	Heart disease (7)	Asthma/COPD (7)	Asthma/COPD (4)	HIV (3)	Asthma/COPD (2)	Asthma/COPD (4)
Sixth most common	Asthma/COPD (4)	HIV (2)	Diabetes (6)	Tuberculosis (3)	Asthma/COPD (3)	Heart disease (2)	Tuberculosis (4)
Seventh most common	Heart disease (2)	Tuberculosis (1)	Heart disease (5)	Heart disease (1)	Tuberculosis (0)	Tuberculosis (1)	Heart disease (3)
Chronic conditions	N positive (% positive)						
Obese	410 (56.9)	43 (43.4)	104 (27.4)	45 (24.1)	98 (25.7)	62 (14.7)	762 (34.8)
Hypertension	271 (37.6)	50 (50.5)	161 (42.5)	54 (28.9)	105 (27.5)	63 (14.9)	704 (32.2)
HIV	187 (26)	2 (2.0)	56 (14.8)	34 (18.2)	13 (3.4)	56 (13.3)	348 (15.9)
Diabetes	69 (9.6)	8 (8.1)	21 (5.5)	7 (3.7)	31 (8.1)	17 (4.0)	153 (7.0)
Asthma/COPD	25 (3.5)	10 (10.1)	27 (7.1)	7 (3.7)	11 (2.9)	10 (2.4)	90 (4.1)
Tuberculosis	48 (6.7)	1 (1.0)	29 (7.7)	6 (3.2)	0 (0.0)	3 (0.7)	87 (4.0)
Heart disease	13 (1.8)	7 (7.1)	17 (4.5)	1 (0.5)	18 (4.7)	8 (1.9)	64 (2.9)
Any chronic condition	617 (85.7)	73 (73.7)	256 (67.5)	114 (61)	193 (50.5)	163 (38.6)	1416 (64.7)
Number of preexisting chronic conditions							
0	103 (14.3)	26 (26.3)	123 (32.5)	73 (39.0)	189 (49.5)	259 (61.4)	773 (35.3)
1	315 (43.8)	37 (37.4)	141 (37.2)	79 (42.2)	130 (34)	115 (27.3)	817 (37.3)
2	210 (29.2)	26 (26.3)	80 (21.1)	31 (16.6)	47 (12.3)	41 (9.7)	435 (19.9)
3	82 (11.4)	8 (8.1)	28 (7.4)	3 (1.6)	12 (3.1)	6 (1.4)	139 (6.3)
4+	10 (1.4)	2 (2.0)	7 (1.8)	1 (0.5)	4 (1.0)	1 (0.2)	25 (1.1)
Multimorbidity (≥2)	302 (41.9)	36 (36.4)	115 (30.3)	35 (18.7)	63 (16.5)	48 (11.4)	599 (7.4)

Abbreviations: COPD, chronic obstructive pulmonary disease; LIC, low income countries; MICs, middle income countries.

Twenty‐seven percent of the women newly diagnosed with breast cancer presented with multimorbidity (presence of ≥2 additional chronic conditions) ranging from 42% in South Africa to 11% in Uganda (Table 2). Percentages of women with multimorbidity was the highest in South African women both under and over age 50, with 49% of the women ≥50 years presenting with multimorbidity in addition to breast cancer. Women from lower MICs Zambia and Nigeria and LIC Uganda had the least prevalence of multimorbidity in both age groups (Figure 1).

**FIGURE 2:**
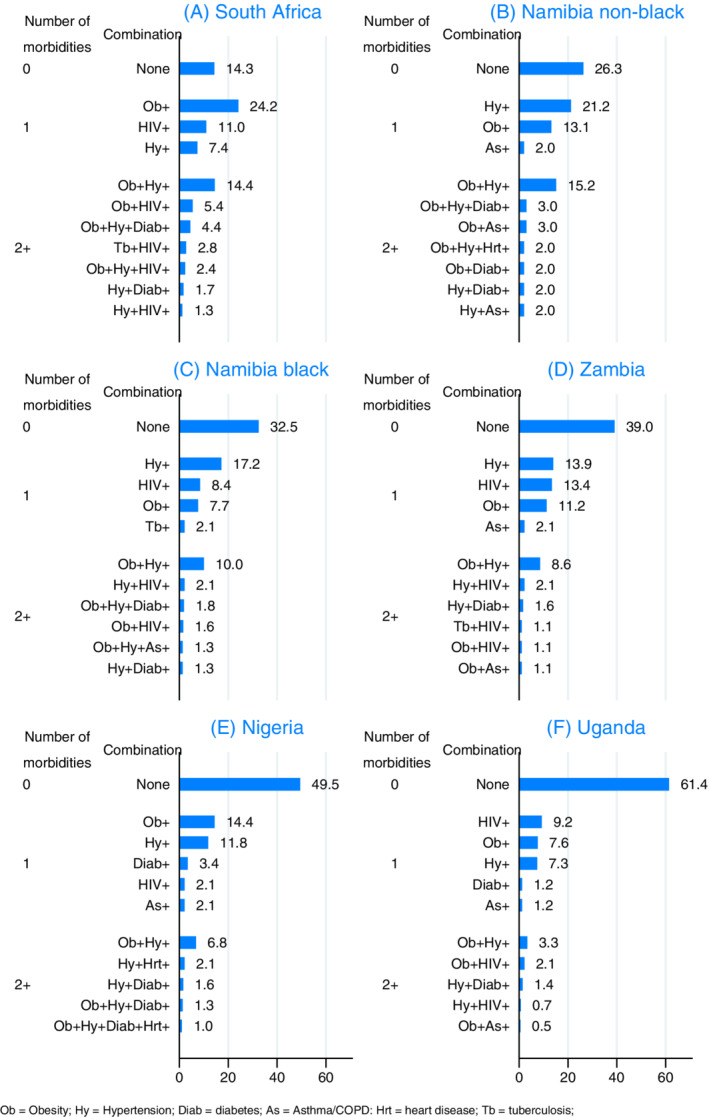
Profile of the combination of seven preexisting morbidities among breast cancer patients in the African Breast Cancer—Disparities in Outcomes study [Color figure can be viewed at wileyonlinelibrary.com]

With seven conditions included, of a possible 128 (2^7^) morbidity combinations, 57 combinations were present among the 2189 women and only 16 combinations were present in at least 5 women. The profile and prevalence of these combinations for the 11 most common combinations in each population group is shown in Figure 2 (ie, no preexisting morbidities and 10 combinations with at least one preexisting morbidities). Where multimorbidity was present, obesity and hypertension were the dominant combination, affecting 15% of Namibian non‐black and 14% of South African women. The only other combination affecting at least 5% of breast cancer patients in a given group was obesity and HIV in South Africa.

### 3.2 Determinants of chronic conditions

Associations of sociodemographic factors with each chronic condition are presented in Table 3. In the mutually adjusted analysis of all countries combined, older women were significantly more likely to be obese, hypertensive, diabetic and have heart disease and less likely to be living with HIV. Of the three measures of socioeconomic status, SEP was most frequently associated with the presence of a chronic condition. Women with higher SEP (7‐9 (high) and 4‐6 (medium)) were more likely to be obese and diabetic compared to women with low SEP (0‐3). High SEP women were also more likely to have hypertension compared to those with low SEP (OR: 1.89, 95% CI 1.33‐2.59). Associations of age and SEP with the two communicable diseases, tuberculosis and HIV, tended to be in the opposite direction to those for the aforementioned NCDs. Notably, women with medium SEP were less likely to have HIV compared to the women with low SEP. Those employed were more likely to be obese while level of education was not significantly associated with any chronic condition except in Nigeria where those with secondary education and above were more likely to have heart disease (result not shown).

**TABLE 3:** Associations of sociodemographic characteristics with each chronic conditions, among breast cancer patients in sub‐Saharan Africa, African Breast Cancer—Disparities in Outcomes study

	Non‐communicable diseases					Communicable diseases		
Sociodemographic characteristics	Obesity (BMI ≥ 30.0 kg/m^2^)	Hypertension	Diabetes	Asthma/COPD	Heart disease	HIV positive	Tuberculosis	NCDs combined
	OR (95% CI)	OR (95% CI)	OR (95% CI)	OR (95% CI)	OR (95% CI)	OR (95% CI)	OR (95% CI)	OR (95% CI)
Age (10 year increase)	1.09 (1.01‐1.18)^a^	1.98 (1.81‐2.17)^b^	1.51 (1.32‐1.74)^b^	1.08 (0.91‐1.29)	1.69 (1.37‐2.09)^b^	0.64 (0.58‐0.71)^b^	0.97 (0.81‐1.15)	1.62 (1.50‐1.76)^b^
Marital status								
Not married	1.00 (Ref)	1.00 (Ref)	1.00 (Ref)	1.00 (Ref)	1.00 (Ref)	1.00 (Ref)	1.00 (Ref)	1.00 (Ref)
Married	1.25 (1.02‐1.54)^a^	1.07 (0.87‐1.33)	1.39 (0.97‐2.00)	0.90 (0.57‐1.42)	0.80 (0.45‐1.43)	0.51 (0.39‐0.67)^b^	0.62 (0.36‐1.03)	1.02 (0.84‐1.25)
Highest level of education								
Primary education and below	1.00 (Ref)	1.00 (Ref)	1.00 (Ref)	1.00 (Ref)	1.00 (Ref)	1.00 (Ref)	1.00 (Ref)	1.00 (Ref)
Secondary education and above	1.01 (0.79‐1.29)	1.05 (0.82‐1.36)	0.77 (0.51‐1.16)	1.03 (0.60‐1.77)	1.06 (0.56‐2.02)	1.18 (0.86‐1.61)	1.13 (0.65‐1.97)	1.04 (0.82‐1.33)
Employment status								
Unemployed	1.00 (Ref)	1.00 (Ref)	1.00 (Ref)	1.00 (Ref)	1.00 (Ref)	1.00 (Ref)	1.00 (Ref)	1.00 (Ref)
Employed	1.51 (1.17‐1.97)^a^	0.98 (0.74‐1.29)	1.11 (0.68‐1.82)	1.16 (0.62‐2.16)	0.71 (0.32‐1.56)	0.88 (0.65‐1.18)	0.90 (0.51‐1.59)	1.22 (0.95‐1.58)
SEP								
Low (0‐3)	1.00 (Ref)	1.00 (Ref)	1.00 (Ref)	1.00 (Ref)	1.00 (Ref)	1.00 (Ref)	1.00 (Ref)	1.00 (Ref)
Medium (4‐6)	1.93 (1.54‐2.40)^b^	1.20 (0.95‐1.51)	1.77 (1.16‐2.69)^a^	1.85 (1.09‐3.15)^a^	1.08 (0.58‐1.99)	0.60 (0.46‐0.79)^b^	1.35 (0.83‐2.20)	1.76 (1.42‐2.18)^b^
High (7‐9)	3.05 (2.22‐4.18)^b^	1.86 (1.33‐2.59)^b^	3.25 (1.88‐5.62)^b^	1.74 (0.86‐3.51)	1.59 (0.72‐3.52)	0.65 (0.41‐1.02)	1.26 (0.55‐2.86)	2.75 (2.02‐3.75)^b^
Country								
Uganda	1.00 (Ref)	1.00 (Ref)	1.00 (Ref)	1.00 (Ref)	1.00 (Ref)	1.00 (Ref)	1.00 (Ref)	1.00 (Ref)
Namibia Black	1.93 (1.34‐2.78)^b^	3.76 (2.61‐5.41)^b^	1.06 (0.54‐2.09)	2.71 (1.28‐5.76)^a^	1.84 (0.76‐4.45)	1.27 (0.84‐1.94)	10.61(3.18‐35.46)^b^	2.60 (1.90‐3.56)^b^
Namibia non‐Black	2.26 (1.35‐3.77)^a^	2.98 (1.73‐5.12)^b^	0.89 (0.35‐2.27)	3.11 (1.17‐8.29)^b^	2.30 (0.71‐7.46)	0.25 (0.10‐1.07)	1.30 (0.13‐13.08)	2.94 (1.72‐5.01)^b^
Nigeria	1.73 (1.19‐2.51)^a^	2.26 (1.54‐3.32)^b^	1.98 (1.04‐3.76)^a^	1.09 (0.44‐2.66)	2.82 (1.15‐6.90)^a^	0.27 (0.14‐0.50)^b^	Omitted	2.23 (1.62‐3.08)^b^
South Africa	9.68 (6.65‐14.07)^b^	2.67 (1.82‐3.92)^b^	2.32 (1.19‐4.55)^a^	1.29 (0.54‐3.06)	0.53 (0.18‐1.57)	2.72 (1.82‐4.08)^b^	8.10 (2.33‐28.12)^a^	6.13 (4.40‐8.54)^b^
Zambia	1.77 (1.12‐2.78)^a^	2.11 (1.33‐3.33)^a^	0.76 (0.30‐1.91)	1.49 (0.54‐4.10)	0.22 (0.03‐1.79)	1.75 (1.06‐2.90)^a^	4.38 (1.06‐18.15)^a^	1.93 (1.30‐2.86)^a^

*Note*: NCDs (obesity, hypertension, diabetes, asthma/COPD, heart disease).

Abbreviations: 95% CI, 95% confidence interval; BMI, body mass index; COPD, chronic obstructive pulmonary disease; NCDs, non‐communicable diseases; OR, odds ratio; SEP, socioeconomic position;.

^a^ Significant at *P* < .05.

^b^ Significant at *P* < .00.

Determinants of the presence of preexisting multimorbidities mirror the associations mentioned earlier, particularly for the NCDs, as the copresence of obesity and hypertension was the most prevalent combinations. Adjusting for age and country site higher SEP compared to low SEP was associated with greater risk for multimorbidity at breast cancer diagnosis (Supplementary Table 1).

### 3.3 Influence of preexisting morbidities on stage at diagnosis

**FIGURE 3:**
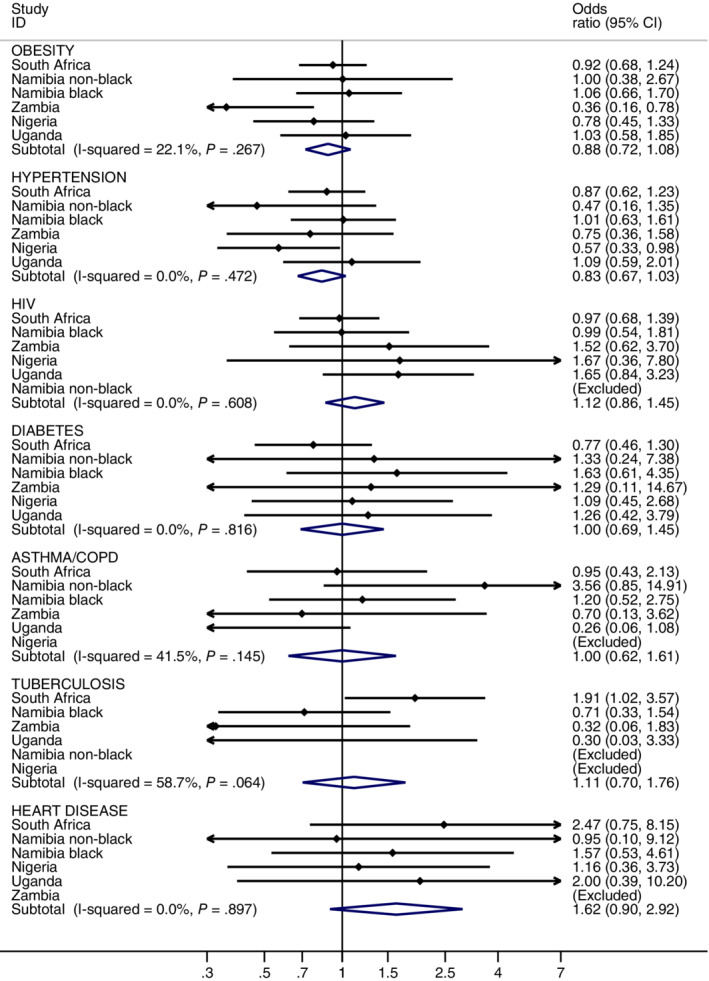
Odds ratios of women being diagnosed with advanced stage breast cancer (stages III and IV) associated with each of the seven chronic conditions examined by sub‐Saharan Africa countries in the African Breast Cancer—Disparities in Outcomes study [Color figure can be viewed at wileyonlinelibrary.com]

Among 2066 women with known stage at diagnosis, investigations of whether preexisting morbidities affected stage at diagnosis are illustrated in Figure 3 for each site, and results of the ABC‐DO wide‐pooled analysis are shown in Table 4 and Supplementary Table 2. Most site‐specific associations were not significant or suggestive, with the following exceptions. Adjusting for age at diagnosis, obese women from Zambia (OR: 0.36, 95% CI 0.16‐0.78) and hypertensive women from Nigeria (OR: 0.57, 95% CI 0.33‐0.98) were less likely to be diagnosed with advanced stage breast cancer compared to their country counterparts without these conditions. In contrast, among South African women, those women who had tuberculosis were more likely to be diagnosed with advanced stage breast cancer than those without such a history (OR: 1.91, 95% CI 1.02‐3.57). Nigeria, Zambian and Ugandan women who were HIV infected were more likely to be diagnosed with advanced stage breast cancer than their HIV‐negative counterparts, though these associations were not significant and were not present in all settings (Figure 3).

**TABLE 4:** Multiple logistic regression models of chronic conditions and presence of multimorbidity (≥2 chronic conditions) as predictors of advanced stage breast cancer (stages III and IV) in women newly diagnosed with breast cancer in sub‐Saharan Africa, African Breast Cancer–Disparities in Outcomes study

Chronic conditions	Odds ratio	95% CI	*P* value
Obese			
No	1.00	Reference	**.049**
Yes	0.72	0.52‐0.99	
Hypertension			
No	1.00	Reference	**.015**
Yes	0.65	0.45‐0.92	
HIV			
Negative	1.00	Reference	.331
Positive	0.85	0.58‐1.22	
Any chronic condition			
No	1.00	Reference	.120
Yes	1.30	0.92‐1.84	
Multimorbidity			
<2 chronic conditions	1.00	Reference	.092
≥2 chronic conditions	1.34	0.95‐1.89	

*Note*: Multiple regression model (adjusted for age, country and HIV). Obesity, hypertension, any chronic condition and multimorbidity were the only variables significant at *P* < .01 on bivariate analysis and are evaluated in the multivariate analysis. Variables significant at *P* < .05 shown in boldface.

Thus, meta‐analytic estimates across all population groups suggested no association of tuberculosis, asthma/COPD, diabetes and HIV with stage at diagnosis, consistent with the pooled analyses in Table 4. However for hypertension, in meta‐analyses, there was a suggestion of a 17% (−3% to 33%) relative reduction in advanced stage breast cancer associated with having this condition, which increased to a 35% (1%‐55%) reduction in pooled analyses adjusted for HIV. Similarly for obesity, the meta‐analysis was suggestive of 12% (95% CI −8% to 28%) reduced odds of advanced stage at diagnosis, which became significant in pooled analyses (28% reduction (1%‐48%). Despite these individual conditions of obesity and hypertension being the most common multimorbidity combination, the overall effect of multimorbidity on advanced stage at cancer diagnosis was borderline suggestive of a positive association (OR: 1.34, 95% CI 0.95‐1.89) (Table 4).

## 4 DISCUSSION

### 4.1 Summary of findings

In this large cohort of breast cancer patients in five SSA countries, the prevalence of at least one of seven preexisting chronic conditions (obesity, hypertension, diabetes, heart disease, asthma/COPD, HIV infection and tuberculosis) was high (65%) and 27% of women had at least two preexisting chronic conditions (multimorbidity). Obesity and hypertension were the two most frequent conditions present, both individually and as a combination among women with multimorbidities, particularly affecting older women and groups with higher socioeconomic status both at the intracountry individual level and, reflecting epidemiologic transitions, at national levels. HIV ranked third in most countries except in non‐black Namibian women and in West African Nigeria. The management of breast cancer in SSA thus needs to consider this high percentage of NCDs, alone or in combination with HIV. Nevertheless, none of the seven chronic conditions investigated were positively associated with having advanced stage breast cancer at diagnosis, with the exception of obesity and hypertension, which was associated with having early stage breast cancer. The latter findings point to an opportunity to further increase early diagnosis, in an attempt to counterbalance any survival disadvantage among these women.

### 4.2 Major prevalent chronic conditions: in the population as risk factors for breast cancer and impact on prognosis

Multimorbidity prevalence closely tracked the wealth index of the countries under investigation and the SEP scores of the women under investigation. Women from higher MICs with more advanced westernised lifestyle transitions had the greater multimorbidity burden (South Africa (42%) followed by Namibia) than those from lower middle‐income Zambia and Nigeria and low‐income Uganda. These findings were in keeping with ranges of 14% to 68% reported from other studies.^12^, ^25^, ^26^ With 27% of our patients presenting with multimorbidity (≥2 chronic conditions), SSA is facing a significant challenge of multimorbidity comprising both infectious diseases combined with the rapidly growing NCD prevalence as countries transition to westernised lifestyles. Hypertension ranked as the most common chronic condition in most countries except in South Africa where obesity ranked first, ranging from 15% in LIC Uganda to 51% among MIC non‐black Namibian women. Hypertension is a public health challenge in Africa with 46% of its population aged 25 years and over living with hypertension.^27^ Hypertension might be positively linked to risk of breast cancer especially in postmenopausal women,^28^ hence its high prevalence among our women is particularly worrying. More critically, during the therapeutic phase, chemotherapy can worsen hypertension and cause cardiomyopathy.^29^

Obesity, a known risk factor for breast cancer in postmenopausal women,^30^ has also been associated with worse breast cancer outcomes for women of all ages.^18^ Obesity was commonly reported in our study though with high variation across countries in various stages of lifestyle transition. South Africa has the highest prevalence with 57% of the women obese while Uganda had the lowest prevalence (15%). The emerging prevalence of obesity in SSA has been largely attributed to the rising empowerment of women, sedentary lifestyles, economic development and urbanisation,^31^ ultimately leading to nutritional transition. Obesity at postmenopausal ages is a driver of increasing breast cancer incidence rates, thus in this instance the rising obesity rates are fuelling more cases with poorer prognosis. Diabetes with prevalence ranging from 4% in Uganda to 10% in South Africa is also a known risk factor for breast cancer in postmenopausal women,^32^ it is also associated with worse survival with up to an increased risk of 52% in overall mortality.^33^

The high burden of HIV among particularly our southern African women in our study is of continued concern with HIV now regarded as a chronic infectious disease, though the general consensus is that there is no significant link between HIV and risk of breast cancer;^34^ however, studies have reported poorer prognosis in HIV‐positive patients with breast cancer.^4^, ^35^

### 4.3 Determinants of chronic conditions

As expected, most chronic conditions were more prevalent in older women, a finding consistent with previous studies in SSA.^12^, ^36^ We also found that women with higher SEP were more likely to present with obesity, hypertension and diabetes in contrast to findings from other mainly HIC studies where multimorbidity was more prevalent at lower SEP.^26^, ^37^, ^38^ However our findings were consistent with other studies from LMICs for multimorbidity,^25^ overweight/obesity, hypertension and diabetes.^36^ These findings perhaps reflects westernised lifestyle and dietary transitions first affecting higher SEP groups with the capital needed to purchase and live modern lifestyles.^39^

### 4.4 Impact on stage

It is known that advanced cancer stage at diagnosis is affected by chronic conditions;^8^, ^9^, ^10^ however, some studies have consistently demonstrated earlier stage at diagnosis with chronic conditions.^11^, ^12^, ^40^ In our study, we found no significant association between multimorbidity and advanced stage at breast cancer diagnosis. Women who had hypertension at diagnosis of breast cancer were more likely to have early stage breast cancer, perhaps due to their regular access to the health system facilities for other conditions.^36^, ^37^ Our patients that were obese were less likely to present with advanced stage breast cancer, this is in contrast to studies suggesting that higher BMI is associated with a more advanced stage of breast cancer at diagnosis.^41^, ^42^ Our finding could be attributable to reverse causality given the 59% of late‐stage tumour in the cohort.

### 4.5 Strengths and limitations

Our study benefitted from a diversity of countries across SSA, a wide range of morbidities included, a strong SSA‐perspective on the selection of morbidities, heterogeneity in sociodemographic factors, measured BMI, tested HIV in South Africa and a prospective design. However, the sample was tertiary hospital‐based patients, and many breast cancer patients in SSA may never reach this level of the health system. Further limitations were noted: most of these chronic conditions were self‐reported; hence, we could have underestimated the prevalence of multimorbidity especially in women with lower SEP. In SSA, low socioeconomic status is associated with lower access to care and treatment^43^ and thus there could be lower rates of diagnosis of these chronic conditions in women with lower SEP.

### 4.6 Implications on therapeutic management

The implication of multimorbidity in women with breast cancer is profound including the high cost of care^44^ and poor quality of life.^45^ Breast cancer patients with multimorbidity are less likely to receive surgery,^46^ less likely to receive adjuvant chemotherapy,^47^ more likely to receive a reduced dose and less likely to complete chemotherapy treatment when initiated,^48^ and less likely to initiate timely radiotherapy after surgical treatment.^49^ Multimorbidity may cause higher rates of adverse effects of treatment^50^ affecting overall goal of care. It has been associated with an increased risk of cardiotoxicity, immunosuppression during chemotherapy and radiotherapy for HIV‐positive women.^51^ Furthermore, at the health system level, the management of multimorbidity is much more complicated and demanding for the patients and the health system, requiring an integrated approach and long‐term care.^52^ To effectively address these changing needs, countries in SSA require detailed surveillance on chronic condition trends to assist in developing models of care appropriate for LMICs.

### 4.7 Conclusion

There is a high prevalence of chronic conditions and multimorbidity in our breast cancer patients and sociodemographic factors play a major role in its determinants in SSA. With increasing life expectancy, the rapidly increasing multimorbidity burden in SSA is of great concern for its under resourced healthcare services. SSA studies of the impact of these morbidities on survival and therapeutic management are needed.

## CONFLICT OF INTEREST

The authors declare no conflict of interest.

## DISCLAIMER

Where authors are identified as personnel of the International Agency for Research on Cancer/World Health Organization, the authors alone are responsible for the views expressed in this article and they do not necessarily represent the decisions, policy or views of the International Agency for Research on Cancer/World Health Organization.

## ETHICS STATEMENT

ABC‐DO was approved by ethics committees at each recruitment institution: IARC (IEC 13‐19, IEC15‐18), the London School of Hygiene and Tropical Medicine (6459), University of Witwatersrand, South Africa (M150345), Council for Science and Technology (HS 1588) and the Ministry of Health and Social Services of Namibia (17/3/3), University of Zambia Biomedical Research Ethics Committee (004‐08‐15), Federal Medical Centre Owerri, Abia State University Teaching Hospital and Uganda National Council for Science and Technology (HS 1588). All participants provided written informed consent.

## Supporting information


**Supplementary Table 1** Sociodemographic, geographical and behavioural factors associatedwith multimorbidity(at least 2 of obesity, hypertension, HIV, diabetes, asthma/COPD, heart disease, tuberculosis in addition to breast cancer)in women newly diagnosed with breast cancer in Sub‐Saharan Africa, ABC‐DO study.
**Supplementary Table 2** Association between individual chronic condition and multimorbidity(≥2 chronic conditions) with stage at diagnosis in women newly diagnosed with breast cancer in Sub‐Saharan Africa, ABC‐DO study.

## Data Availability

Data for this study contain confidential patient information. The datasets analysed during the current study are available from the corresponding author on reasonable request.
