# Supplementary material for: Preexisting morbidity profile of women newly diagnosed with breast cancer in sub‐Saharan Africa: African Breast Cancer—Disparities in Outcomes study
Source: Int J Cancer. 2020 Nov 25;148(9):2158–70. doi: 10.1002/ijc.33387 (PMC8129872; doi:10.1002/ijc.33387)
Supplement: Supplementary file 1 — Supplementary Table 1 Sociodemographic, geographical and behavioural factors associatedwith multimorbidity(at least 2 of obesity, hypertension, HIV, diabetes, asthma/COPD, heart disease, tuberculosis in addition to breast cancer)in women newly diagnosed with breast cancer in Sub‐Saharan Africa, ABC‐DO study. Supplementary Table 2 Association between individual chronic condition and multimorbidity(≥2 chronic conditions) with stage at diagnosis in women newly diagnosed with breast cancer in Sub‐Saharan Africa, ABC‐DO study. [file IJC-148-2158-s001.pdf]

Pre-existing morbidity profile of women newly diagnosed with breast cancer in sub-Saharan Africa: African Breast Cancer—Disparities in Outcomes (ABC-DO) study

Oluwatosin A. Ayeni, MBChB, M.Sc, Shane A. Norris, PhD, Maureen Joffe, PhD, Herbert Cubasch, MD, Moses Galukande, MD, Annelle Zietsman, MD, Groesbeck Parham, MD, Charles Adisa, MD, Angelica Anele, MD, Joachim Schüz, PhD, Benjamin O. Anderson, MD, Milena Foerster, PhD, Isabel dos Santos Silva, PhD, Valerie A. McCormack, PhD.

Table of Contents

Supplementary table 1..3
Supplementary table 2..4
African Breast Cancer—Disparities in Outcomes (ABC-DO) study questions on chronic conditions...5

Supplementary table 1

Socio-demographic, geographical and behavioural factors associated with multimorbidity (at least 2 of obesity, hypertension, HIV, diabetes, asthma/COPD, heart disease, tuberculosis in addition to breast cancer) in women newly diagnosed with breast cancer in Sub-Saharan Africa, ABC-DO study.

	**Multimorbidity**			
	**Total**	**No (< 2 chronic conditions)**	**Yes (≥ 2 chronic conditions)**	**Adjusted analysis**
	N=2189 (%)	N=1590 (%)	N=599 (%)	OR (95% CI)
**Age in years, mean ± SD**	51.6±13.9	49.9±14.0	56.4±12.2	**1.03 (1.02-1.04)[b]**
**Marital status**				
Married	1217 (55.6)	859 (54)	358 (59.8)	1.00 (Reference)
Not married	972 (44.4)	731 (46)	241 (40.2)	1.11 (0.90-1.37)
**Highest level of education**				
Primary education and below	827 (37.8)	608 (38.2)	219 (36.6)	1.00 (Reference)
Secondary education and above	1362 (62.2)	982 (61.8)	380 (63.4)	1.12 (0.88-1.42)
**Employment status**				
Unemployed	782 (35.7)	510 (32.1)	272 (45.4)	1.00 (Reference)
Employed	1407 (64.3)	1080 (67.9)	327 (54.6)	1.07 (0.82-1.40)
**SEP**				
Low (0-3)	916 (41.8)	725 (45.6)	191 (31.9)	1.00 (Reference)
Medium (4-6)	928 (42.4)	625 (39.3)	303 (50.6)	**1.45 (1.16-1.82)[b]**
High (7-9)	345 (15.8)	240 (15.1)	105 (17.5)	**2.15 (1.57-2.96)[b]**
**Country**				
Uganda	422 (19.3)	374 (23.5)	48 (8)	1.00 (Reference)
Namibia Black	379 (17.3)	264 (16.6)	115 (19.2)	**3.09 (2.11-4.50)[b]**
Namibia Non-Black	99 (4.5)	63 (4)	36 (6)	**3.62 (2.16-6.06)[b]**
Nigeria	382 (17.5)	319 (20.1)	63 (10.5)	**1.53 (1.02-2.31)[a]**
South Africa	720 (32.9)	418 (26.3)	302 (50.4)	**4.93 (3.52-6.92)[b]**
Zambia	187 (8.5)	152 (9.6)	35 (5.8)	**1.75 (1.08-2.82)[a]**
**Ever consumed alcohol?**				
No	1313 (60)	932 (58.6)	381 (63.6)	1.00 (Reference)
Yes	876 (40)	658 (41.4)	218 (36.4)	1.08 (0.87-1.34)
**Ever smoked?**				
No	2031 (92.8)	1490 (93.7)	541 (90.3)	1.00 (Reference)
Yes	158 (7.2)	100 (6.3)	58 (9.7)	1.03 (0.71-1.50)

OR (Odds ratio), 95% CI (95% confidence interval), SD (standard deviation), SEP (Socio-economic position), COPD (Chronic obstructive pulmonary disease, [a] Significant at $p<0.05$, [b] Significant at $p<0.001$. ORs adjusted for age and count

Supplementary table 2

Association between individual chronic condition and multimorbidity (≥2 chronic conditions) with stage at diagnosis in women newly diagnosed with breast cancer in Sub-Saharan Africa, ABC-DO study.

	**Total**	**Early stage (Stages I & II)**	**Advanced stage (Stages III & IV)**	**P value**
**Chronic conditions**	**N=2066 (%)**	**N=853 (%)**	**N=1213 (%)**	
**Obese**				
No	1334 (64.6)	512 (60.0)	822 (67.8)	**<0.001**
Yes	732 (35.4)	341 (40.0)	391 (32.2)	
**Hypertension**				
No	1394 (67.5)	535 (62.7)	859 (70.8)	**<0.001**
Yes	672 (32.5)	318 (37.3)	354 (29.2)	
**HIV status**				
Negative	1735 (84.0)	719 (84.3)	1016 (83.8)	0.746
Positive	331 (16.0)	134 (15.7)	197 (16.2)	
**Diabetes**				
No	1924 (93.1)	790 (92.6)	1134 (93.5)	0.440
Yes	142 (6.9)	63 (7.4)	79 (6.5)	
**Tuberculosis**				
No	1980 (95.8)	817 (95.8)	1163 (95.9)	0.912
Yes	86 (4.2)	36 (4.2)	50 (4.1)	
**Asthma/COPD**				
No	1978 (95.7)	816 (95.7)	1162 (95.8)	0.883
Yes	88 (4.3)	37 (4.3)	51 (4.2)	
**Heart diseases**				
No	2003 (97.0)	832 (97.5)	1171 (96.5)	0.193
Yes	63 (3.0)	21 (2.5)	42 (3.5)	
**Any chronic condition**				
No	714 (34.6)	260 (30.5)	454 (37.4)	**0.001**
Yes	1352 (65.4)	593 (69.5)	759 (62.6)	
**Multimorbidity**				
<2 chronic conditions	1491 (72.2)	588 (68.9)	903 (74.4)	**0.006**
≥2 chronic conditions	575 (27.8)	265 (31.1)	310 (25.6)	

***analysis limited to 2066 women with known stage at diagnosis. P value significant at <0.1 shown in bold face.
COPD (Chronic obstructive pulmonary disease),

African Breast Cancer—Disparities in Outcomes (ABC-DO) study questions on chronic conditions.

"Have you ever been diagnosed with any of the following (Read each item - tick each one that applies)?

[ ] high blood pressure/hypertension

[ ] heart disease

[ ] diabetes (insulin)

[ ] diabetes (tablets)

[ ] anaemia

[ ] COPD/chronic bronchitis/emphysema

[ ] asthma

[ ] hepatitis B or C

[ ] TB

[ ] other chronic infection (e.g. malaria)

[ ] other cancer

[ ] HIV, and are you currently taking antiviral medication: [ ] yes [ ] no

[ ] any other disease ____

[ ] no, none of these."

For anthropometry, we had:

[Please measure the participant's standing height in centimetres] _____________________________cm

[Please measure the participant's weight in kilograms] ____kg
