# Supplementary material for: Preexisting morbidity profile of women newly diagnosed with breast cancer in sub-Saharan Africa: African Breast Cancer—Disparities in Outcomes study
Source: Int J Cancer. Author manuscript; Available in PMC 2021 May 18. (PMC8129872; doi:10.1002/ijc.33387)
Supplement: Supplementary file 1 [file NIHMS1698300-supplement-Supp_Table_1.pdf]

Supplementary table 1: Socio-demographic, geographical and behavioural factors associated with multimorbidity (at least 2 of obesity, hypertension, HIV, diabetes, asthma/COPD, heart disease, tuberculosis in addition to breast cancer) in women newly diagnosed with breast cancer in Sub-Saharan Africa, ABC-DO study.

	Multimorbidity			
	**Total**	**No (< 2 chronic conditions)**	**Yes (≥ 2 chronic conditions)**	**Adjusted analysis**
	N=2189 (%)	N=1590 (%)	N=599 (%)	OR (95% CI)
**Age in years, mean ± SD**	51.6±13.9	49.9±14.0	56.4±12.2	**1.03 (1.02-1.04)[b]**
**Marital status**				
Married	1217 (55.6)	859 (54)	358 (59.8)	1.00 (Reference)
Not married	972 (44.4)	731 (46)	241 (40.2)	1.11 (0.90-1.37)
**Highest level of education**				
Primary education and below	827 (37.8)	608 (38.2)	219 (36.6)	1.00 (Reference)
Secondary education and above	1362 (62.2)	982 (61.8)	380 (63.4)	1.12 (0.88-1.42)
**Employment status**				
Unemployed	782 (35.7)	510 (32.1)	272 (45.4)	1.00 (Reference)
Employed	1407 (64.3)	1080 (67.9)	327 (54.6)	1.07 (0.82-1.40)
**SEP**				
Low (0-3)	916 (41.8)	725 (45.6)	191 (31.9)	1.00 (Reference)
Medium (4-6)	928 (42.4)	625 (39.3)	303 (50.6)	**1.45 (1.16-1.82)[b]**
High (7-9)	345 (15.8)	240 (15.1)	105 (17.5)	**2.15 (1.57-2.96)[b]**
**Country**				
Uganda	422 (19.3)	374 (23.5)	48 (8)	1.00 (Reference)
Namibia Black	379 (17.3)	264 (16.6)	115 (19.2)	**3.09 (2.11-4.50)[b]**
Namibia Non-Black	99 (4.5)	63 (4)	36 (6)	**3.62 (2.16-6.06)[b]**
Nigeria	382 (17.5)	319 (20.1)	63 (10.5)	**1.53 (1.02-2.31)[a]**
South Africa	720 (32.9)	418 (26.3)	302 (50.4)	**4.93 (3.52-6.92)[b]**
Zambia	187 (8.5)	152 (9.6)	35 (5.8)	**1.75 (1.08-2.82)[a]**
**Ever consumed alcohol?**				
No	1313 (60)	932 (58.6)	381 (63.6)	1.00 (Reference)
Yes	876 (40)	658 (41.4)	218 (36.4)	1.08 (0.87-1.34)
**Ever smoked?**				
No	2031 (92.8)	1490 (93.7)	541 (90.3)	1.00 (Reference)
Yes	158 (7.2)	100 (6.3)	58 (9.7)	1.03 (0.71-1.50)

OR (Odds ratio), 95% CI (95% confidence interval), SD (standard deviation), SEP (Socio-economic position), COPD (Chronic obstructive pulmonary disease, [a] Significant at $p<0.05$, [b] Significant at $p<0.001$. ORs adjusted for age and coun
