# Supplementary material for: Preexisting morbidity profile of women newly diagnosed with breast cancer in sub-Saharan Africa: African Breast Cancer—Disparities in Outcomes study
Source: Int J Cancer. Author manuscript; Available in PMC 2021 May 18. (PMC8129872; doi:10.1002/ijc.33387)
Supplement: Supplementary file 2 [file NIHMS1698300-supplement-Supp_table_2.pdf]

Supplementary table 2: Association between individual chronic condition and multimorbidity (≥2 chronic conditions) with stage at diagnosis in women newly diagnosed with breast cancer in Sub-Saharan Africa, ABC-DO study.

	**Total**	**Early stage (Stages I & II)**	**Advanced stage (Stages III & IV)**	**P value**
**Chronic conditions**	**N=2066 (%)**	**N=853 (%)**	**N=1213 (%)**	
**Obese**				
No	1334 (64.6)	512 (60.0)	822 (67.8)	**<0.001**
Yes	732 (35.4)	341 (40.0)	391 (32.2)	
**Hypertension**				
No	1394 (67.5)	535 (62.7)	859 (70.8)	**<0.001**
Yes	672 (32.5)	318 (37.3)	354 (29.2)	
**HIV status**				
Negative	1735 (84.0)	719 (84.3)	1016 (83.8)	0.746
Positive	331 (16.0)	134 (15.7)	197 (16.2)	
**Diabetes**				
No	1924 (93.1)	790 (92.6)	1134 (93.5)	0.440
Yes	142 (6.9)	63 (7.4)	79 (6.5)	
**Tuberculosis**				
No	1980 (95.8)	817 (95.8)	1163 (95.9)	0.912
Yes	86 (4.2)	36 (4.2)	50 (4.1)	
**Asthma/COPD**				
No	1978 (95.7)	816 (95.7)	1162 (95.8)	0.883
Yes	88 (4.3)	37 (4.3)	51 (4.2)	
**Heart diseases**				
No	2003 (97.0)	832 (97.5)	1171 (96.5)	0.193
Yes	63 (3.0)	21 (2.5)	42 (3.5)	
**Any chronic condition**				
No	714 (34.6)	260 (30.5)	454 (37.4)	**0.001**
Yes	1352 (65.4)	593 (69.5)	759 (62.6)	
**Multimorbidity**				
<2 chronic conditions	1491 (72.2)	588 (68.9)	903 (74.4)	**0.006**
≥2 chronic conditions	575 (27.8)	265 (31.1)	310 (25.6)	

***analysis limited to 2066 women with known stage at diagnosis. P value significant at <0.1 shown in bold face.
COPD (Chronic obstructive pulmonary disease),
